# Primary mucinous ovarian tumors vs. ovarian metastases from gastrointestinal tract, pancreas and biliary tree: a review of current problematics

**DOI:** 10.1186/s13000-021-01079-2

**Published:** 2021-03-11

**Authors:** Pavel Dundr, Naveena Singh, Barbora Nožičková, Kristýna Němejcová, Michaela Bártů, Ivana Stružinská

**Affiliations:** 1 Institute of Pathology, First Faculty of Medicine, Charles University and General University Hospital in Prague, Studničkova 2, 128 00 Prague 2, Czech Republic; 2 Department of Cellular Pathology, Barts Health NHS Trust, Queen Mary University of London, London, UK; 3 Blizard Institute of Core Pathology, Queen Mary University of London, London, UK

**Keywords:** Ovarian tumors, Mucinous carcinoma, Mucinous borderline tumor, Ovarian metastases

## Abstract

**Background:**

Making the distinction between primary mucinous and metastatic ovarian tumors is often difficult, especially in tumors with a primary source from the gastrointestinal tract, pancreas and biliary tree. The aim of the following paper is to provide an overview of the problematics, with a focus on the possibilities of the differential diagnosis at the macroscopic, microscopic and immunohistochemical level.

**Main body:**

The three main aspects of mucinous ovarian tumors are described in detail, including the comparison of the available diagnostic algorithms based on the evaluation of mostly macroscopic features, characterization of the spectrum of microscopic features, and a detailed analysis of the immunophenotype comparing 20 antibodies with the assessment of their statistical significance for differential diagnosis purposes. Specific features, including Krukenberg tumor and pseudomyxoma peritonei, are also discussed.

**Conclusion:**

Despite the growing knowledge of the macroscopic and microscopic features of ovarian mucinous tumors and the availability of a wide range of immunohistochemical antibodies useful in this setting, there still remains a group of tumors which cannot be precisely classified without close clinical-pathological cooperation.

## Introduction

According to historical data, primary mucinous ovarian carcinomas (MC) accounted for about 12% of all ovarian carcinomas [1]. However, some of the historical data was also in contradiction with this high reported incidence of MC, such as the results of the Surveillance Epidemiologic and End Results (SEER) study. In this study, which analyzed data from 1978 to 1998, only 1% (3508 of 35,059) of invasive ovarian cancers were classified as mucinous carcinomas [2]. The results of this study are difficult to interpret, as the percentage of mucinous ovarian carcinomas was extremely low, even when taking into consideration the current knowledge showing lower incidence of MC than historically thought. In approximately the last two decades a lower incidence of MC than which had been previously reported has been confirmed. It has been shown that a significant proportion of MC or mucinous borderline tumors (MBT) of the ovary, which were originally classified as primarily ovarian, are actually of metastatic origin with the primary source located most commonly in the gastrointestinal tract [3]. Based on this data, it seems that MCs are rare tumors, representing approximately 3% of all ovarian cancers. For this reason, any data gained from studies of primary ovarian mucinous tumors which were conducted prior to the 1990s should be viewed with caution, as the likelihood that these studies inadvertently included metastatic tumors is high. However, the distinction between primary mucinous ovarian tumor and a metastasis can be difficult even today. There is still a certain proportion of tumors for which, based on the morphological and immunohistochemical (IHC) features alone, the distinction between a primary and a metastatic tumor is not possible. These tumors require a close clinical-pathological cooperation. The reason for this is that the tumor morphology, IHC features, and even the molecular changes of primary and metastatic tumors may overlap. This applies in particular to cases where metastases to the ovary are clinically apparent before the manifestation of the primary tumor, which may not be detectable at the time of diagnosis, even by the available imaging methods. The approach to the differential diagnosis of these tumors is complex and includes a combination of macroscopic, microscopic, and IHC features. In our review we provide a comprehensive summary of ovarian mucinous tumors focusing on those morphological features which may be helpful in differential diagnosis, including macroscopy, microscopy, and IHC characteristics. The goal of our review is to provide a comprehensive overview of current published data, focusing especially on the algorithmic approach to distinction between primary and metastatic ovarian tumors. Concerning the IHC profile of ovarian mucinous tumors, we performed an extensive literature search in order to prepare a complex, although non-exhaustive review to assess the practical significance of immunohistochemistry in the differential diagnosis of these tumors.

## Methods

An extensive review of the literature on the subject of primary mucinous ovarian cancer and metastatic ovarian tumors was carried out. The data was obtained through a database search using a combination of the MeSH (Medical Subject Headings) terms “mucinous”, “carcinoma”, “ovary”, “ovarian”, “immunohistochemistry”, “primary” and “metastatic“. The data was mined from the PubMed/MEDLINE database covering the time period from 1985 to May 2020. The first search resulted in 11,906 articles. From these the duplicates, articles evidently not relevant to the topic, and case reports were excluded based on the title and revision of the abstract. After that we selected a group of 536 articles which were screened in their entirety in order to select articles relevant to our study. All articles describing the IHC results in a group of ovarian cancer without further specification and possibility of precise allocation of the results to the particular histological type (i.e. mucinous carcinoma) were either excluded, or used only to mine data concerning metastases. Data concerning the endocervical type of mucinous borderline tumors, seromucinous borderline tumors, and endometrioid tumors with mucinous differentiation was excluded. Finally, we selected 49 studies which focus on the problematics of IHC characteristics of primary mucinous ovarian tumors, and/or ovarian metastases and their corresponding primary gastrointestinal tract (GIT), pancreas or biliary tree sources, which are the basis of this review concerning immunohistochemical analyses [4–52]. After excluding both antibodies where the staining results were not available for both primary and metastatic tumors, and antibodies used on a very limited number of cases, 20 primary antibodies were selected for further analysis as the subject of this study. The list of all the included antibodies is provided in Table 1. We should however be aware that different studies may use different clones of the same antibody which may not necessarily result in the same staining. This facts represent a limitation of our review.

**Table 1:** List of immunohistochemical antibodies and their results

	Mucinous ovarian (all)		MC		APE		CRC		Pancreas		Pancreatobiliary		Gastric	
	No. positive (all, %)	No. positive (3+, %)	No. positive (all, %)	No. positive (3+, %)	No. positive (all, %)	No. positive (3+, %)	No. positive (all, %)	No. positive (3+, %)	No. positive (all, %)	No. positive (3+, %)	No. positive (all, %)	No. positive (3+, %)	No. positive (all, %)	No. positive (3+, %)
SATB2	7.2	6.3	8.4	6.4	87	85.1	74.8	76.4	0	NA	0	NA	4.3	NA
CDX2	48.8	22.1	46	25.5	96.6	92.8	93.2	82.1	18.9	NA	31.2	28.6	62.8	17.4
CK7	89.9	86.5	90.3	87.8	26.2	13.3	30.9	5.6	95.1	90	95.5	82.8	67.2	66.7
CK20	68.3	37.1	64.7	40.6	92	77.7	89.8	74.1	57.3	14.3	55.2	22.2	67.6	53.6
ß-catenin	5.8	NA	7.9	NA	NA	NA	57	NA	NA	NA	11.8	NA	20	NA
MUC1	51	13.8	73.7	31.6	NA	NA	39.4	NA	92.2	NA	90.4	NA	54.2	NA
MUC2	47	14.4	44.9	22.2	NA	NA	71.6	26.8	7.8	0	8.7	0	54.2	NA
MUC5AC	84.6	86.5	75.5	55	85.7	71.4	88	6	89.5	91.7	74.8	91.7	76.9	NA
MUC6	49.7	11.2	58.3	13.9	NA	NA	NA	NA	75	NA	55.6	0	11.1	0
DPC4	93.3	98	88.6	87.5	76	NA	80	NA	57.7	47.4	60.3	47.4	96.2	NA
CEA	59	4.6	61.2	4.6	100	NA	89.9	41.5	77.8	NA	77.8	NA	71.4	NA
PAX8	36	9	33.2	8.3	4.6	5.6	0	NA	0	NA	4.2	NA	0	NA
ER	7.9	NA	9.2	NA	6.7	NA	0	NA	0	NA	0	NA	0	NA
PR	9	2.8	8.9	2.8	14.3	NA	0	NA	NA	NA	0	NA	0	NA
CA125	23.7	NA	5.5	NA	0	NA	10.8	0	0	NA	50	NA	0	NA
AMACR	67.2	55.2	67.2	55.2	NA	NA	48.2	34.1	NA	NA	NA	NA	NA	NA
CK19	80	NA	80	NA	NA	NA	40	NA	100	NA	100	NA	NA	NA
villin	41	4.8	NA	NA	95	NA	100	NA	NA	NA	NA	NA	NA	NA
POF1B	21.7	NA	NA	NA	NA	NA	36.4	NA	NA	NA	46.7	NA	46.7	NA
CA19–9	77.6	NA	77.3	NA	NA	NA	60	NA	100	NA	100	NA	NA	NA

For some antibodies the % of 3+ cases is higher than % of all cases. This is due to the fact that the number of 3+ cases was calculated only from the studies in which the semiquantitative IHC results were reported

*NA* not available; *MC* mucinous ovarian carcinoma; *APE* appendix; *CRC* colorectal

Antibodies: *SATB2* AT-rich sequence-binding protein 2; *CDX2* homeobox protein CDX-2; *CK7* cytokeratin 7; *CK20* cytokeratin 20, *MUC1* mucin 1 (also called EMA - epithelial membrane antigen); *MUC2* mucin 2; *MUC5AC* mucin 5 AC; *MUC6* mucin 6; *DPC4* deleted in pancreatic cancer 4 (also called SMAD4 - SMAD family member 4); *CEA* carcinoembryonic antigen; *PAX8* paired box gene 8; *ER* estrogen receptor, *PR* progesterone receptor; *CA125* cancer antigen 125 (also called MUC16 - mucin 16); *AMACR* alpha-methylacyl-CoA racemase; *CK19* cytokeratin 19; *POF1B* premature ovarian failure 1B; *CA19–9* carbohydrate antigen 19–9

The data extracted from these studies was analyzed with a focus on the following parameters: the number of all cases, the number of positive and negative cases, and the extent of positivity (divided into categories 1+, 2+, and 3+). However, the criteria for IHC result categorization often differ among the studies analyzed, so only the 3+ positivity was selected for a further sub-analysis (this category was used to cover cases with “diffuse expression” or positive expression in > 50% cells irrespectively of the staining intensity). The percentage of all positive cases and 3+ positive cases was calculated for all the categories when available. For the purposes of the statistical analysis, we compared all mucinous ovarian tumors merged into one category as “primary mucinous tumors” (including mucinous cystadenoma, borderline tumor, and carcinoma) with 5 groups of other tumors. These were classified into: “colorectal carcinoma”, “appendiceal carcinoma”, “pancreatic carcinoma”, “pancreatobiliary carcinoma” and “gastric carcinoma”. This classification was designed due to the wide range of different stratifications of tumors in the selected studies (some of which, for example, reported only joined groups such as “pancreatobiliary”, others used categories like “pancreatic”, “extrahepatic biliary”, etc.). We feel that especially the category of appendiceal tumors deserves a separate assessment for appendiceal carcinoma and low grade appendiceal mucinous neoplasm (LAMN). Unfortunately, most of the studies merged these two categories into one group of tumors, and as such precise allocation of the result to either appendiceal carcinoma or LAMN was not possible. For studies using the joined groups of tumors, the results for both extraovarian primary tumors (i.e. colorectal carcinoma) and metastatic tumors (i.e. ovarian metastasis of colorectal carcinoma) were merged. The results reported only in broader categories, such as “upper GI tract”, were excluded from our review. The number of positive/negative cases (recorded values) was statistically compared between the “primary mucinous tumors” and the five groups of metastatic tumors for all of the 20 antibodies, using the Pearson Chi-square test or Fisher’s Exact test, depending on the expected values [53, 54]. Statistical analyses were performed using the software TIBCO Statistica 13.3.0. All tests were two-sided and a *p*-value of less than 0.05 was considered as significant.

The data concerning the algorithmic approach to differential diagnosis is based on the results of 11 studies [3, 13, 16, 55–62]. The general macroscopic and microscopic features of primary and metastatic ovarian tumors were extracted from several studies, including the review articles.

### Macroscopic and microscopic features of mucinous ovarian tumors

**Fig. 1:**
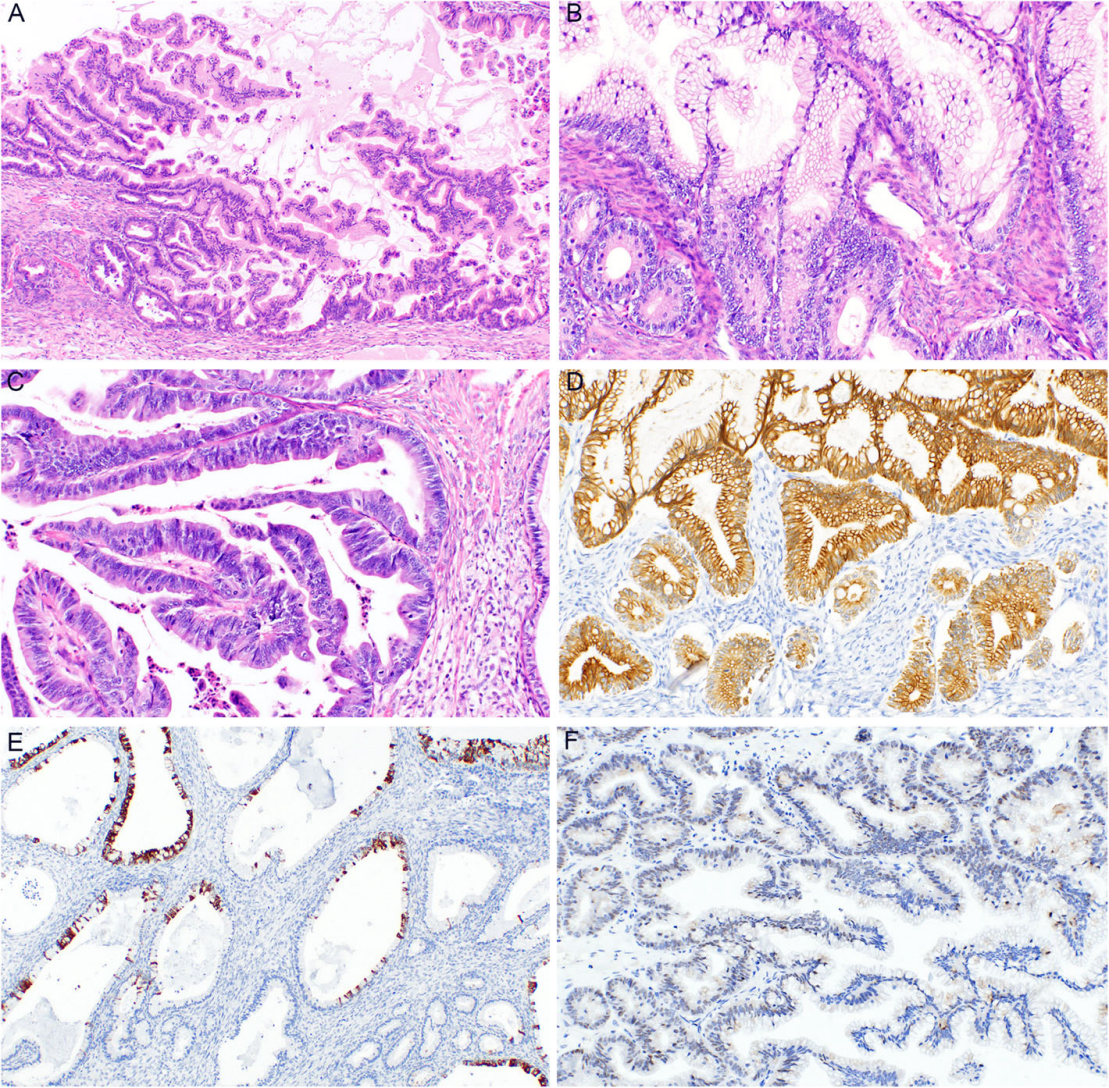
Mucinous borderline tumor (MBT). Intracystic epithelial proliferation of gastrointestinal-type epithelium with villoglandular arrangement (**a**) (H&E, 100x). Tumor structures showing increased proliferative activity in the crypts with mitoses and mild nuclear atypia, whereas other parts of the villoglandular structures consist of more mature cells (**b**) (H&E, 200x). MBT with intraepithelial carcinoma consisting of tumor cells with marked nuclear atypia and increased mitotic activity (**c**) (H&E, 200x). Diffuse immunohistochemical expression of cytokeratin 7 (**d**) (200x) and focal expression of cytokeratin 20 (**e**) (200x). MBT with focal, mostly weak expression of PAX8 (**f**) (200x)

**Fig. 2:**
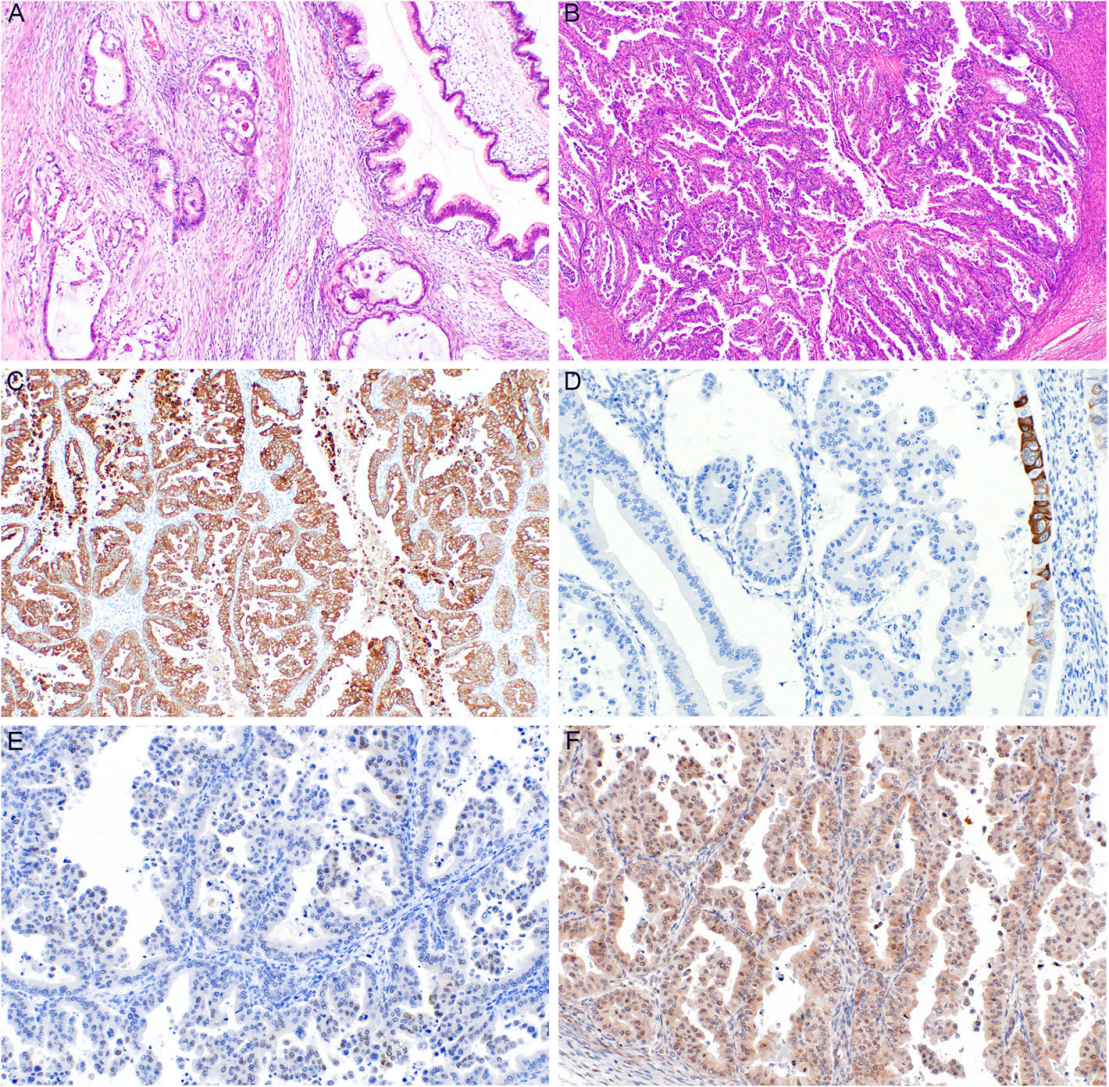
Mucinous carcinoma of the ovary (MC). Infiltrative type of invasion with glandular and cribriform areas (**a**) (H&E, 100x). Expansile type of invasion with areas of complex glandular proliferation (**b**) (H&E, 40x). Diffuse expression of cytokeratin 7 (**c**) (100x) and focal expression of cytokeratin 20 (**d**) (200x). Focal and weak expression of PAX8 (**e**) (200x). Tumor cells showing retained expression of DPC4 (**f**) (200x)

**Fig. 3:**
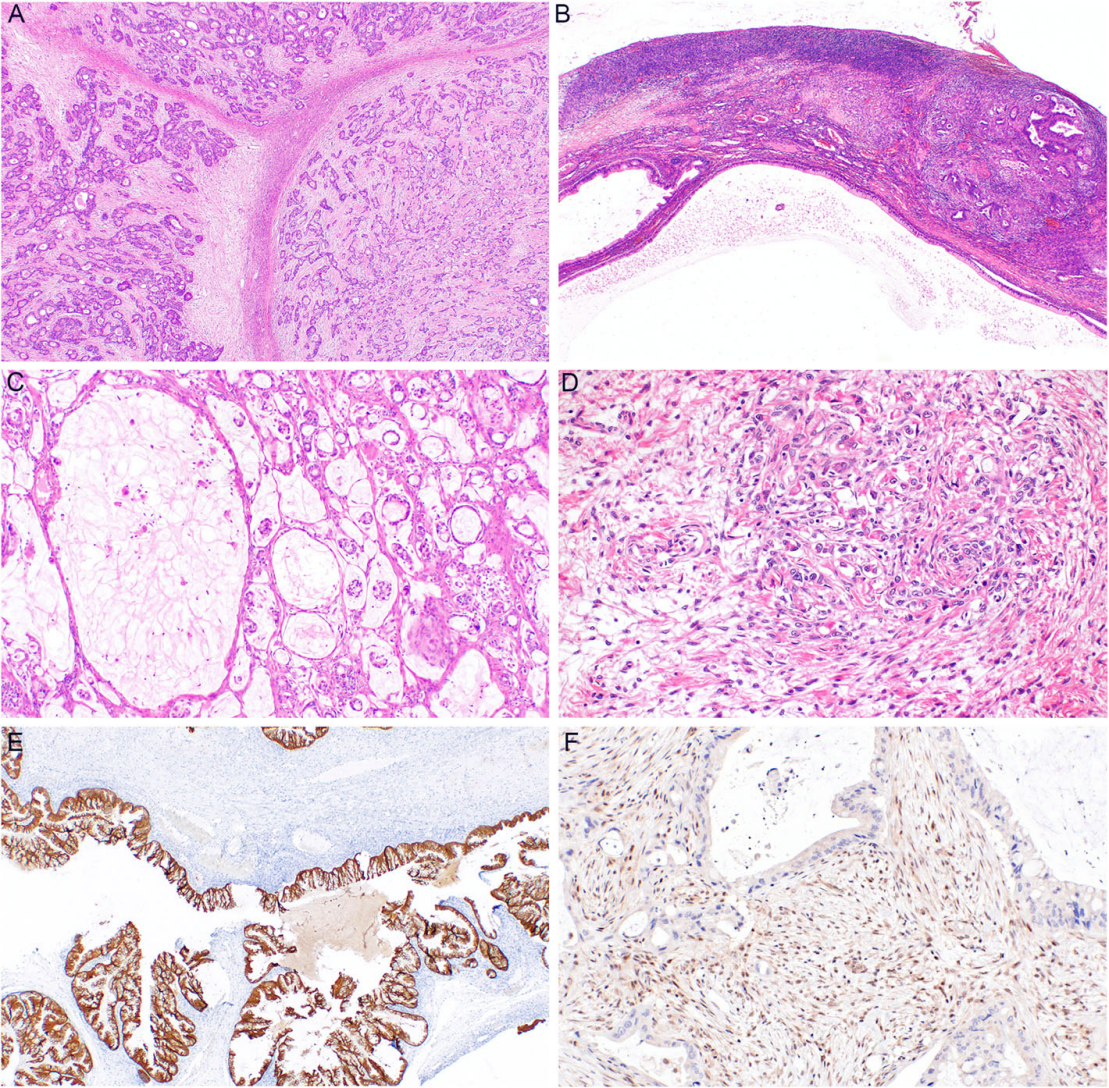
Ovarian metastases from GIT tumors. Metastasis of colorectal carcinoma showing nodular arrangement (**a**) (H&E, 40x). Metastasis of pancreatic carcinoma with areas of benign appearance and a nodule of infiltrative growth (**b**) (H&E, 40x). Gallbladder metastasis with infiltrative growth of irregular glands (**c**) (H&E, 100x). Metastases of gastric carcinoma with typical appearance of Krukenberg tumor with “pseudosarcomatous” stromal reaction (**d**) (200x). Ovarian metastasis of appendiceal low grade mucinous neoplasm showing positivity of cytokeratin 20 (**e**) (40x). Pancreatic metastasis showing loss of DPC4 expression (**f**) (200x)

Both primary and metastatic mucinous ovarian tumors are characterized by a number of macroscopic and microscopic features, which may be helpful in their differential diagnosis (Figs. 1, 2 and 3). However, these are not entirely specific and a subset of metastatic tumors may sometimes mimic primary ovarian tumors [3, 8, 55, 63, 64]. In general, most primary ovarian mucinous carcinomas are large, unilateral tumors that are cystic, multilocular, with intact smooth surface, without any nodularities. Cystic and solid areas in these tumors are evenly distributed throughout the tumor. Areas with features of benign mucinous cystadenoma or MBT are common. The predominant type of invasion is usually expansile, with the infiltrative type of invasion being absent or only focal in most cases. On the contrary, metastatic tumors are often smaller, bilateral, and involve the ovarian surface or superficial cortex. The nodularity is usually visible macroscopically, however, even cases without macroscopically distinct nodules usually show these at a microscopic level, where they are characterized by aggregates (groups, nodules) of tumor cells surrounded by normal ovarian stroma, especially in the superficial cortex. Another feature which seems to be relatively specific for ovarian metastasis of LAMN is gross mucinous multinodular appearance [65]. Metastatic tumors also often show a predominantly infiltrative type of invasion. However, some metastases (especially of pancreatobiliary tract malignancies) are known to often mimic primary ovarian tumors [8, 17, 66, 67]. These tumors commonly show areas of benign or MBT appearance. Some tumors even consist only of these areas and do not show any areas of invasive growth (so-called paradoxical maturation). Metastatic tumors of pure MBT appearance are typically characterized by more pronounced nuclear atypia, and the lesions mimic an MBT with intraepithelial carcinoma. Moreover, even metastatic tumors can be large, unilateral, and even without surface involvement. According to our review, in studies in which this data was available, unilateral tumors ≥10 cm represent 15% of all metastatic tumors (29/190 cases; range 5–19.8%). These tumors are particularly difficult to diagnose as metastatic, especially in cases when they represent the first manifestation of a hitherto clinically occult primary extraovarian disease.

A summary of the morphological features is given in Table 2, although there is no single feature which would allow for a clear distinction between a primary and a metastatic tumor. For example laterality, which is cited as one of the key features when distinguishing between primary (mostly unilateral) and metastatic (more often bilateral) tumors, is by itself a highly unreliable feature. As with other parameters, historical data concerning mucinous carcinomas should be viewed with caution. For example, in a study published in 2005 analyzing the data from the Surveillance Epidemiologic and End Results (SEER) program (which included data from 22,328 women with ovarian cancer without central evaluation from the period between 1992 and 2000), MC was reported as bilateral in 355/1665 cases (21.3%) regardless of the disease stage [68]. The high percentage of primary bilateral tumors suggests that some of these tumors were probably of a metastatic origin. Based on our analysis of the data, which we performed using the data mined from 26 studies dealing with this issue, bilaterality was found in 9.4% of all primary mucinous ovarian tumors (104/1105), in 10.3% of MC (95/918) and in 49.7% of metastases (1176/2365), regardless of the primary source [3, 17, 20, 23, 34, 38, 46, 47, 55, 60–62, 66, 67, 69–80]. However, when evaluating colorectal cancer metastases separately, bilaterality was found in only 25.8% of cases (25/97). Still, the percentage of bilateral primary ovarian tumors (10.3% of primary MC) seems to be too high and the possibility that some studies may be biased by metastatic ovarian tumors misclassified as primary tumors cannot be excluded with certainty. Tumor laterality alone is not sufficient enough to distinguish between primary and metastatic tumors, and a careful evaluation which takes into consideration other tumor features is necessary. However, laterality is still one of the key parameters evaluated in the algorithmic approaches (usually in combination with the size of the tumor), which are discussed in detail below.

**Table 2:** List of morphological features of primary and metastatic ovarian carcinomas

Favor primary	Favor metastatic	Unhelpful features
smooth capsule	involvement of surface and superficial cortex	gross cysts
evenly distributed cystic and solid areas (no discrete nodularity)	nodular pattern (gross and/or microscopic) including gross mucinous multinodular appearance	gross solid, papillary, hemorrhagic areas
areas of MBT and/or mucinous cystadenoma	areas of MBT and/or mucinous cystadenoma less frequent, commonly associated with high grade nuclear atypia	nature of the content of the cysts
expansile invasion	infiltrative (destructive) invasion	pseudomyxoma ovarii
complex papillary pattern	bilaterality	cribriform, villous, or solid growth
size > 10 cm (> 15 cm)	hilar involvement	goblet cells
associated teratoma or Brenner tumor	single cell invasion	tumor grade
microscopic cystic glands	signet ring cells	focal areas resembling colonic carcinoma
	vascular invasion	
mural nodule	microscopic mucus on the surface	
unilateral	pseudomyxoma peritonei	
low stage	intraperitoneal spread	
low age	colloid morphology	
	established history of cancer	

*MBT* mucinous borderline tumor

One of the features which deserves a special recognition because of its high specificity for metastatic origin is the presence of signet ring cells. Tumors containing signet ring cells are, in keeping with stringent diagnostic criteria regardless of their primary source, referred to as Krukenberg tumors. By definition, a Krukenberg tumor is a tumor in which, in addition to signet ring cells, the tumor stroma is often present in the form of a “sarcomatoid” stromal reaction [81, 82]. Krukenberg, after whom the tumor is named, hypothesized that it was an unusual type of a mucin-producing fibrosarcoma. However, subsequent studies have shown that these tumors are indeed metastatic, most commonly with a primary source in gastric cancer and less often in colorectal cancer, with other locations being implicated only rarely [83]. Stromal reaction is a reactive process which often takes on a sarcomatoid appearance, and as such can cause difficulties in differential diagnosis. However, literature data on this tumor is severely influenced by inconsistencies in the approach to its classification. The term Krukenberg tumor is sometimes used for any adenocarcinoma metastasizing to the ovary, regardless of the presence of signet ring cells and stromal response. In some studies the tumors are even termed as Krukenberg tumors regardless of their primary source. Sometimes the term Krukenberg tumor is used only for gastrointestinal tract metastases. Nevertheless, we should be aware that signet ring cells may rarely occur even in primary ovarian mucinous carcinomas, so this feature is also not entirely specific for metastastic origin [84, 85]. However, signet ring cell histology compared to non-signet ring cell histology showed a specificity of 99.7% for indicating metastastic origin (with the sensitivity reaching only 12.0%), with a positive predictive value for metastasis of 98.4% [62].

Another feature which is highly (although not entirely) specific for metastatic tumors is the presence of pseudomyxoma peritonei (PMP) [86]. The histogenesis of mucinous ovarian tumors is multifactorial, but current knowledge suggests that some tumors are of a teratomatous origin, whereas others may arise from mucinous metaplasia, probably in the form of Walthard’s nests or in Brenner tumors [87]. Tumors arising in a teratoma, which represents 3–8% of ovarian mucinous tumors, show somewhat different morphological, immunohistochemical and other features, and represent a heterogeneous group of tumors [45]. They include tumors which in many respects mimic intestinal (appendicular) tumors, most likely arising from teratomatous lower intestinal tract tissue, and tumors which are close in morphology and immunophenotype to tumors from the upper gastrointestinal or pancreatobiliary tract. Moreover, a possible origin in sinonasal-type teratoma tissue has been suggested for some intestinal type adenocarcinomas, characterized by an immunophenotype indicative of upper gastrointestinal tract (with CK7 positivity and CK20 positivity or negativity) [45]. In contrast to tumors of non-teratomatous origin, which typically show diffuse CK7 expression and a variable CK20 expression, teratomatous tumors are more heterogeneous and include CK7 negative and CK20 positive cases (which is an immunophenotype typical for colorectal carcinoma).

Another difference from tumors of non-teratomatous origin is the frequent association with pseudomyxoma peritonei in teratoma-associated lesions. In a study of 42 teratoma-associated mucinous tumors, 10 were associated with PMP (1 MA, 6 MBT, and 3 MC) [43]. However, 4 cases classified as PMP in this study showed only the presence of acellular mucin, the other 3 contained epithelial structures with features of a low-grade mucinous tumor, and the last 3 displayed carcinomatous characteristics. Changes evaluated as pseudomyxoma ovarii were also present in 8 of these tumors. An appendectomy was performed in 7 patients, all of which showed no tumor structures in the appendix. Compared to PMP associated with a low grade mucinous tumor of the appendix (LAMN), the prognosis of PMP in teratoma-associated lesions appears to be better (except in cases of peritoneal carcinomatosis associated with MC). However, the data is relatively limited in this respect. Macroscopically, the mucus of ovarian origin is much thinner compared to the thicker, jelly-like mucus of PMP associated with appendiceal tumors [88].

Generally, in the case of ovarian tumors showing morphology and immunophenotype characteristic for the lower gastrointestinal tract, one should consider the possibility of a minor teratomatous component which may have not been detected on sampling, and some authors recommend performing an extensive material re-examination, in addition to correlation with clinical data [45].

### Algorithmic approach to differential diagnosis of ovarian mucinous tumors

We have found 11 studies focusing on the algorithmic approach to differential diagnosis between primary and metastatic mucinous ovarian cancer [3, 13, 16, 55–62]. Generally, the idea of an algorithmic approach is based on different gross features between primary and metastatic ovarian tumors, including especially the size and laterality. The most common approach is based on the assumption that a primary tumor is unilateral, in combination with a size criterion (≥ 10; 12; 13 or 15 cm), and any and all other tumors are considered as metastatic. In some studies this algorithm is modified by excluding tumors with signet ring cell component, which are in most cases metastatic. Additionally, some algorithms also take into account other features, including the age of the patients and some immunohistochemical results, such as PAX8, DPEP1 (dipeptidase 1; a zinc-dependent metallopeptidase involved in glutathione metabolism which is commonly expressed in colorectal carcinoma), CK7 and CDX2 expression [13, 58]. Table 3 shows our summary of the algorithms used in 9 studies, in which the available data allowed for a comparison between the primary and metastatic cases (including the accuracy, sensitivity, specificity, negative predictive value (NPV), and positive predictive value (PPV)). Two studies were excluded from the analysis - one because it analyzed only metastatic cases, and the other one because it did not include the combination of size and laterality [16, 55]. In certain aspects the results of all studies are similar, in that irrespectively of the algorithm used they generally show the same trend – if the criteria for primary (metastatic) tumor are stricter, then the increased specificity is associated with decreased sensitivity. The summary of the results from all the included studies showed the following: accuracy (mean 83.5%, median 83.8%, range 71.3–96.1%, SD 5.8); sensitivity for the identification of a primary tumor (mean 84.1%, median 82.2%, range 56.3–100%, SD 14.3); specificity for the identification of a primary tumor (mean 82.8, median 81, range 72.2–94.7, SD 6.6).

**Table 3:** Algorithmic approach to the differential diagnosis of ovarian mucinous tumors

Study	N primary	N metastatic	Algorithm	Accuracy %	Sensitivity^f^ %	Specificity^f^ %	PPV	NPV
Seidman et al. (Ref. [3])	12	40	≥ 10 cm, unilateral	90.0	75.0	94.7	81.8	92.3
			≥ 10 cm, unilateral, excl. Signet ring cell	83.0	–	–	–	–
Khunamorpong et al.^a^ (Ref. [56])	16	52	≥ 10 cm, unilateral, excl. Signet ring cell	83.8	81.3	84.3	61.9	93.6
			≥ 15 cm, unilateral, excl. Signet ring cell	83.8	56.3	92.3	69.2	87.3
Yemelyanova et al. (Ref. [57])	52	142	≥ 10 cm, unilateral	83.5	100	77.5	61.9	100
			≥ 10 cm, unilateral, excl. Signet ring cell	87.3	100	81	72.2	100
			≥ 12 cm, unilateral	85.6	100	80.3	65	100
			≥ 12 cm, unilateral, excl. Signet ring cell	83.2	100	75.7	65	100
			≥ 13 cm, unilateral	86.1	98.1	81.7	66.2	99.1
			≥ 13 cm, unilateral, excl. Signet ring cell	84.4	98.1	78.3	67.1	98.9
Okamoto et al. (Ref. [58])	58^c^	36	≥ 10 cm, unilateral	71.3^b^	–	–	–	–
			DPEP1, CK7, CDX2, size^d^	93.3				
Jung et al. (Ref. [59])	19	91	≥ 10 cm, unilateral	82.7	94.7	80.2	50	98.6
			≥ 10 cm, unilateral, excl. Signet ring cell	80.6	94.7	77.7	51.4	98.3
			≥ 13 cm, unilateral	87.3	78.9	89	60	95.3
			≥ 13 cm, unilateral, excl. Signet ring cell	84.9	78.9	86.5	60	94.1
			≥ 15 cm, unilateral	89.1	64.8	93.4	68.4	93.4
Maeda-Taniguchi et al. (Ref. 60])	51	22	≥ 10 cm, unilateral	75.3	70.6	86.4	92.3	55.9
Hu et al. (Ref. [13])	47	18	primary: ≥ 10 cm, unilateral, PAX8 +/− meta: PAX8 - and bilateral (any size) or unilateral < 10 cm	86.2	91.5	72.2	89.6	87
			≥ 10 cm, unilateral	75.4	–	–	–	–
Hu et al. (Ref. [61])	61	68	primary: ≥ 13 cm unless bilateral or has surface nodules meta: < 13 cm unless unilateral	96.1	–	–	–	–
Simons et al. (Ref. [62])	735	1018	algorithm based on histology (signet ring cells), laterality, size and age^e^	77.1	59	90.1	81.1	75.3
			≥ 10 cm, unilateral	76.6	82.2	72.5	68.3	84.9
			≥ 13 cm, unilateral	77.2	73.6	79.9	72.5	80.7

^a^ Excluding metastatic cases with endometrioid-like and signet ring cell features (only tumors with “mucinous” morphology left)

^b^ Data from only 87 primary and metastatic cases, further details not available

^c^ Incl. 13 cases on endocervical type MBT

^d^ Criteria for size are not clear, for the details see ref. [58]

^e^ For details see ref. [62]

^f^ Calculated for primary ovarian tumors

- data not available

In summary, algorithms by themselves are not sufficient for the definite distinction between primary and metastatic ovarian tumors, but they do provide important information which can be used in combination with microscopic features and immunohistochemical profile in order to classify most tumors with high accuracy. In one study, the suggested algorithm was intentionally optimized for high sensitivity for metastatic tumors, as the authors emphasized that a misdiagnosis of a metastatic tumor as a primary MC has greater consequences for the patient [62]. However, the achieved high sensitivity for mOC (90.1%) was associated with low specificity (59.0%). In another study, a combination of CDX2, CK7 and DPEP1 showed an accuracy of 97% (56/58 tumors) for the detection of primary MC, and accuracy of 100% (16/16 tumors) for the detection of mCRC. However, the reported accuracy for metastases from the upper GIT reached only 56% (9/16 cases) [58]. Interestingly, when the algorithm results were stratified according to the primary source of metastases, in one study the success ratio showed much better results for pancreatobiliary tract neoplasms than for colorectal carcinoma [57].

### Immunohistochemistry

The results for all antibodies are summarized in Table 1. The statistical significance of each antibody for the differential diagnosis is summarized in Table 4. The results show that there are in fact several antibodies, which reached statistical significance when comparing primary mucinous tumors and metastatic / extraovarian tumors. However, statistical significance alone does not necessarily equate to practical usefulness. Generally, there is no single antibody upon which the decision concerning the primary source of the tumor may be based with certainty.

**Table 4:** Statistical significance of immunohistochemical results between primary mucinous ovarian tumors and metastatic tumors

	Appendix	Colorectum	Pancreas	Pancreatobiliary	Gastric
SATB2	**< 0.001**	**< 0.001**	***0.015***	**0.003**	*0.456*
CDX2	**< 0.001**	**< 0.001**	**< 0.001**	< 0.001	0.076
CK7	**< 0.001**	**< 0.001**	0.094	**0.028**	**< 0.001**
CK20	**< 0.001**	**< 0.001**	**0.031**	**0.001**	0.904
B-catenin	NA	**< 0.001**	NA	*0.316*	*0.232*
Villin	**< 0.001**	***0.039***	NA	NA	NA
MUC1	NA	0.119	**< 0.001**	< 0.001	0.763
MUC2	NA	< 0.001	**< 0.001**	< 0.001	0.499
MUC5AC	NA	< 0.001	0.277	0.013	*0.276*
MUC6	NA	NA	**0.021**	0.526	***0.036***
POF1B	NA	0.126	NA	*0.098*	*0.098*
Ca19.9	NA	*0.249*	***0.007***	***0.007***	NA
DPC4	***0.014***	**< 0.001**	**< 0.001**	< 0.001	*0.735*
CEA	**0.002**	**< 0.001**	*0.316*	*0.316*	0.224
PAX8	**< 0.001**	**< 0.001**	*0.055*	**0.001**	< 0.001
ER	NA	***0.032***	NA	*0.370*	*0.210*
PR	NA	*0.583*	NA	NA	NA
CA125	*0.354*	**0.006**	NA	NA	***0.006***
AMACR	NA	**0.025**	NA	NA	NA
CK19	NA	0.170	*0.076*	*0.076*	NA

*P*-values are based on Pearson Chi-square test or Fisher Exact test (italics). Significant p-values are indicated in **bold**

In the differential diagnosis between primary and metastatic ovarian mucinous tumors it is necessary to take into account the origin of the mucinous tumor because, as it has already been mentioned, some tumors arising in a teratoma may be indistinguishable from metastatic tumors on the IHC level [43, 45]. In cases of other mucinous tumors, the use of IHC depends on the differential diagnosis in question.

In the differential diagnosis with metastases from the lower GIT (colorectal and appendiceal carcinoma) a combination of CK7, CK20, CDX2, SATB2, and PAX8 is often used. Especially for CK7 and CK20 it is appropriate to evaluate not only the presence of positivity, but also its extent and the relationship of mutual expression of these 2 antibodies. Primary mucinous ovarian tumors express CK7 in about 90% of cases, and the expression is almost always diffuse (about 85% of tumors express CK7 in more than 50% of tumor cells, however, the criteria for diffuse expression vary among studies) [32, 33, 51, 89]. CK20 expression is also relatively common in primary ovarian mucinous tumors (about 65–70% of cases), but diffuse expression is found only in about 40% of cases. When evaluating the coordinate expression of CK7/CK20, regardless of its extent, primary ovarian mucinous tumors are positive for both markers in 67% of cases, CK7 positive / CK20 negative in 26% of cases, and CK7 negative / CK20 positive in only 7% of cases. However, primary mucinous tumors arising in a teratoma are CK7 negative / CK20 positive in 50% of cases [45]. CDX2 expression occurs in 49% of cases of primary tumors, but strong expression is observed in only 26% [18, 27, 90, 91]. SATB2 is a marker which is significantly more specific for colorectal cancer than CDX2 [4–7, 10–12, 92]. In primary ovarian mucinous tumors SATB2 is expressed only in about 8% of cases. In contrast, PAX8 expression is reported in about 35% of primary cases, although it can be relatively weak and only focal. In comparison to primary mucinous ovarian tumors, colorectal and appendiceal carcinoma metastases show CK7 expression in 31 and 26% of cases respectively, although the expression is diffuse in only 6 and 13% of cases. In contrast, CK20 is positive in 90 and 92% of cases, and almost always diffuse. CDX2 expression is positive in about 93% of colorectal and 97% of appendiceal cancers, and is almost always diffuse. SATB2 positivity is reported in 87% of appendiceal and 75% of colorectal tumors, and is almost always diffuse as well. According to our review, PAX8 is positive in about 5% of appendiceal cancers, while in colorectal cancer the expression of PAX8 has not been reported at all. Certain studies also evaluated the coordinate expression for some antibodies, which is an approach which seems to be more beneficial for the purposes of differential diagnosis. The most commonly used combination is CK7 and CK20, but combinations of other antibodies have been reported as well, including PAX8, CK7, CK20, CDX2 and SATB2. The results concerning the coordinate expression extracted from 10 studies are summarized in Table 5 [5, 9, 16, 20, 22, 38, 43, 45, 51, 93]. However, with the exception of the CK7/CK20 combination, the data is rather limited. Briefly, the coordinate expression of CK7/CK20 in ovarian metastases is significantly different compared to primary mucinous tumors. The expression of both markers is reported in about 22% of appendiceal and 11% of colorectal cancers, and most these tumors (78 and 79%) are CK7 negative / CK20 positive. According to our review, CK20 negative / CK7 positive staining pattern occurs in 0% of appendiceal tumors and about 3% of colorectal tumors. Negativity of both markers is reported in 0% of appendiceal and 6% of colorectal tumors. In summary, immunohistochemical examination can be helpful in the differential diagnosis between primary ovarian mucinous tumors and metastases from the “lower” GIT, but the use of a panel of antibodies and the correct interpretation of their results is crucial.

**Table 5:** Immunohistochemical results of the coordinate expression

		Mucinous (all) (%)	Mucinous teratoma (%)	APE (%)	CRC (%)	Pancreas (%)	Pancreatobiliary (%)	Gastric (%)
CDX2/CK20	+/+	50	–	90	–	–	–	–
	−/+ OR +/−	29.5	–	10	–	–	–	–
	−/−	20.5	–	0	–	–	–	–
SATB2/CK20	+/+	0	–	80	–	–	–	–
	−/+ OR +/−	72.2	–	20	–	–	–	–
	−/−	27.8	–	0	–	–	–	–
SATB2/PAX8	+/+	1.5	–	–	–	–	–	–
	+/−	10.2	–	–	–	–	–	–
	−/+	40	–	–	–	–	–	–
	−/−	48.3	–	–	–	–	–	–
CK7/CK20	+/+	67.2	27	22.2	11.3	70.4	66.6	31.6
	+/−	26	16.2	0	3.2	25.9	28.2	21
	−/+	6.8	50	77.8	79	0	2.6	31.6
	−/−	0	6.8	0	6.5	3.7	2.6	15.8
CK7/CDX2	+/+	37.2	–	–	–	–	–	–
	+/−	53.8	–	–	–	–	–	–
	−/+	9	–	–	–	–	–	–
	−/−	0	–	–	–	–	–	–

*APE* appendiceal carcinoma; *CRC* colorectal carcinoma; − not available

Metastases from the pancreatobiliary tract represent probably the most problematic category with regard to possible confusion with primary ovarian tumors [8, 17, 66, 94]. Regarding the IHC examination, the antibodies listed in the differential diagnosis between primary ovarian mucinous tumors and metastases of the “lower” GIT are practically useless in the context of pancreatobiliary neoplasms. The expression of CK7 and CK20 is almost identical in both groups of tumors. Although the expression of CDX2 is slightly more common in primary ovarian mucinous tumors, in practice this difference is difficult to utilize (49% positivity in primary ovarian tumors vs. 19% in pancreatic cancers and 31% in pancreatobiliary cancers). SATB2 expression is not reported in pancreatobiliary tumors; however, its expression in primary ovarian mucinous tumors is rare (7%), which significantly limits its practical use. Of the other markers, the expression of PAX8, DPC4, and CK17 seems to be useful for the purposes of differential diagnosis. PAX8 is reported to be positive in 36% of primary ovarian mucinous tumors and in 4% of pancreatobiliary tumors. However, the sensitivity of this marker is low. A loss of DPC4 (SMAD4) expression occurs in 53% of pancreatic and only about 5–10% of primary ovarian mucinous tumors [28, 36, 37, 95]. According to the literature, cytokeratin 17 seems to be negative in ovarian mucinous tumors and positive in 27–83% of metastatic pancreatic carcinomas, but the data is very limited [94, 96].

The most problematic (on the IHC level) is the distinction between primary ovarian mucinous tumor and gastric adenocarcinoma metastasis [75, 97, 98]. Morphologically, some of the metastases have a signet ring cell component, or consist only of these elements, which is a feature strongly suggestive against the diagnosis of a primary ovarian tumor. However, it is very complicated to make the distinction between primary and metastatic tumors based on IHC results. The expression of CK7, CK20, CDX2 and SATB2 is very similar in both groups and therefore these markers cannot be used. Practically useful antibodies are mainly represented by PAX8 (positive in 35% of primary mucinous tumors vs. 0% of gastric cancers) and CA125 (positive in 24% of primary mucinous tumors vs. 0% of gastric cancers). However, these antibodies are only significant when the staining result is positive and their sensitivity is low. A minority of primary ovarian mucinous tumors (< 10%) may also weakly express ER and PR. The expression of ER/PR is not reported in gastric cancer, but again it is a marker with very low sensitivity.

### Incidence of metastases and their primary source

The frequency of ovarian metastases from the GIT may vary, but according to the literature ovarian metastases occur during the course of the disease in 2% of patients with colorectal cancer and 2.9% of patients with gastric cancer [69, 75, 78, 97, 99–103]. Concerning ovarian metastases, we have analyzed the data from 13 studies, which were used to mine the information on primary source [3, 28, 46, 60, 80, 104–111]. Moreover, 5 of these studies also provided data concerning the percentage of metastatic ovarian cancer from all (primary and metastatic) ovarian tumors. Based on these 5 studies, we were able to determine that out of the 14,060 cases of ovarian cancers 656 cases (4.7%) were metastases. Based on all of the 13 studies, the most common primary source was colorectal carcinoma (32%), followed by breast carcinoma (15.4%), endometrial carcinoma (12.9%), gastric carcinoma (9.2%), appendiceal carcinoma (6.7%), uterine cervix carcinoma (2.4%), pancreatic carcinoma (2.2%), small intestine carcinoma (1.6%), and carcinoma of the gallbladder and biliary tract (1.5%). Other tumors such as lung carcinoma, skin tumors, kidney cancer, and esophageal carcinoma each accounted for less than 1%. For the entire group of metastatic tumors the primary source was unknown in 12.2%.

## Discussion

Metastatic ovarian tumors are common, however, according to published literature their frequency covers a wide range and represents 5–30% of all ovarian carcinomas [29, 46, 109, 112]. According to our review, 4.7% of ovarian cancers were metastatic. Most of the ovarian metastases have a primary source in GIT tumors, which accounted for 53.2% of tumors according to our review. The distinction between primary ovarian MC and ovarian metastasis from another primary source is important and has a direct influence on patient’s treatment and prognosis. Although this topic has been given relatively a lot of attention in the literature, the historical data is equivocal due to the fact that some tumors previously classified as primary MC were in fact metastases [3]. Today, the knowledge that certain metastatic tumors can mimic primary mucinous ovarian tumors is well recognized [81, 82]. The situation is also complicated by the fact that a subset of ovarian metastases represents the first manifestation of a hitherto unrecognized extraovarian disease. Despite the increasing knowledge on this issue, including advancements in the array of methodological options, currently there still are no methods or algorithms which would allow us to distinguish between primary and metastatic tumors with certainty and this remains problematic area as is acknowledged in the new 5th edition of WHO Classification of Female genital organs tumors as well [113]. For a proportion of tumors the primary source of the tumor remains unclear even after exhaustive comprehensive examinations. These cases must be addressed within multidisciplinary teams, but in rare cases, ambiguities persist even after a comprehensive clinico-pathological evaluation. The metastatic nature of a certain ovarian tumor may in some cases become apparent only after several months, due to the manifestation of the previously occult, unrecognized extraovarian tumor [114].

Another approach which can be theoretically helpful in the differential diagnosis of ovarian mucinous tumors is their molecular characterization. However, according to current knowledge the aberration occurring in ovarian mucinous tumors are not specific, which prevents the use of molecular pathology in this setting. Briefly, the most common aberrations occurring in primary mucinous ovarian carcinoma are mutations of *KRAS* (≈ 55%), *CDKN2A* (≈ 55%, including deletions), *TP53* (≈ 52%), *ARID1A* (≈ 10%), *BRAF* (≈ 8%), and amplification of HER2 (≈ 28%) [115–121]. The aberrations occurring in MBT are similar, with mutations of *KRAS* (≈ 55%), *CDKN2A* (≈ 44%, including deletions), *TP53* (≈ 12%), *BRAF* (≈ 11%), and amplification of HER2 (≈ 10%) [122–126].

The summary of current knowledge shows that most tumors can be reliably classified with respect to the primary source based on the morphological criteria, which include the assessment of macroscopic features, microscopic findings, and immunohistochemical profile of the tumor. However, a minority of tumors, especially from the upper GIT, pancreas and biliary tree, remain problematic in this context [55, 66, 94]. The problem is especially caused by the fact that the morphologic features and immunophenotype of primary mucinous ovarian tumors are not specific, and there are overlapping features with metastases. Although certain features are more common in metastatic tumors (such as surface involvement, nodular growth, hilar involvement and the presence of signet ring cells), they are not specific [17]. Moreover, some metastatic tumors can demonstrate features more common for primary tumors, such as a large size, unilaterality, absence of surface involvement, and areas of benign or borderline appearance (which can rarely be the only morphological pattern). The role of immunohistochemistry in the distinction between primary and secondary ovarian mucinous tumors may be helpful, but we should be aware of its benefits and limitations. First of all there is no single antibody which can differentiate between these tumors with absolute certainty. Immunohistochemical antibodies should be used as a part of a panel, which may be composed as a general one, or be more targeted to a particular possible primary source. Moreover, we should take into account the fact that primary mucinous ovarian tumors which are of a teratomatous origin may share not only the morphology, but also the IHC profile with their intestinal or upper GIT counterpart. It is also important to be aware of coordinate expression, which may be more helpful than isolated assessment of each antibody. Various combinations of PAX8, CK7, CK20, CDX2 and SATB2 have been used with varying success [5, 9, 16, 20, 22, 38, 43, 45, 51, 93]. However, with the exception of the CK7/CK20 combination, the data on their usefulness is rather limited. Based on our review, the immunohistochemistry may be helpful, but contrary to other tumor types, in the distinction between primary mucinous ovarian tumors vs. ovarian metastases from the GIT, pancreas and biliary tree, the role of IHC is rather limited. In general, we should keep in mind the overlapping IHC results between primary and secondary tumors originating from GIT. Also, certain antibodies (such as DPC4) lack sensitivity despite having high specificity.

Additionally, in the differential diagnosis between primary mucinous ovarian tumors and GIT metastasis we should also be aware of the possibility of metastases from cervical adenocarcinoma, which may have morphological features resembling both primary ovarian tumors and GIT metastases [63, 127]. In the case of HPV-associated adenocarcinomas, the best marker for differential diagnosis seems to be p16 [128–130]. Diffuse expression of p16 (> 90% of tumor cells) is very rare in primary mucinous tumors - the largest study focusing on this issue reported diffuse p16 positivity in 5.7% of primary ovarian mucinous carcinomas [131]. In another study the sensitivity of p16 positivity for ovarian metastases of cervical adenocarcinoma reached 100%, with specificity of 98% [128]. However, literary data concerning immunohistochemical profile of HPV-independent related cervical adenocarcinomas of gastric type are limited. According to the largest study focusing on this topic, gastric type cervical adenocarcinomas are positive for CK7 in 100% of cases, CK20 in 49%, CDX2 in 51%, Ca125 in 80%, PAX8 in 68%, ER in 6%, PR in 9% and MUC6 in 81% of cases [132]. According to these results, immunohistochemistry is not very helpful in the differential diagnosis of HPV-independent cervical adenocarcinoma and close clinico-pathological correlation is essential.

The role of a pathologist is crucial not only in determining the final diagnosis (with the use of ancillary methods and clinico-pathological correlations), but also in the perioperative (frozen section) examination. In this setting, the distinction between primary and metastatic tumor can fundamentally modify the surgical procedure, but is usually complicated by limited sampling and the impossibility of utilizing immunohistochemical examinations. In this situation, the benefit of an algorithmic approach combining the macroscopic characteristics of the tumor with histological features can be significant. However, the available algorithms based on the evaluation of macroscopic characteristics of tumors cannot distinguish between primary and metastatic tumors with certainty, despite having a relatively high overall accuracy, sensitivity and specificity. Complementary to the macroscopic and histologic features, it has been shown that the age of the patient represents another important factor possibly playing a role in the differential diagnosis of primary vs. metastatic tumors. In one study from 2011, only 9.1% (2/22) of metastases occur in females younger than 50 years [60]. In the group of primary tumors, however, 49% of patients were younger than 50 years. The factor of age has even been incorporated into the diagnostic algorithm of one recent study [62].

In conclusion, the distinction between primary and secondary ovarian mucinous tumors can be straightforward, but there are still cases in which achieving the correct diagnosis may be complicated. This includes especially two situations which can seriously influence the correct treatment of the patient. In one of them, ovarian metastasis may be misdiagnosed as a primary ovarian tumor due to the pathological features which may simulate primary ovarian tumor. In most such cases the clinical examination reveals the primary tumor in another location. However, in rare cases the primary tumor can be undetectable at the time of diagnosis and will only become clinically apparent later during the course of the disease. This is the reason why each mucinous ovarian tumor, especially carcinoma, should be regarded with caution and thorough clinical examination of the patient with close follow-up is desirable. In the second situation, ovarian tumor with pathological features of metastasis may be the primary manifestation of the disease, which is at the time of diagnosis not detectable despite extensive examination of the patient. In this situation, the pathologist has to pass the information about equivocal features of ovarian tumor suggestive of metastatic origin to the clinicians even in the absence of clinically detectable another potentially primary tumor. The primary tumor in these cases will probably manifest itself later during the disease and a close follow-up of the patient is necessary. According to the current knowledge, the most reliable approach to the diagnosis of ovarian mucinous tumors combines macroscopic, microscopic, and immunohistochemical assessment combined with a close clinico-pathological correlation. However, we should be aware that despite all the effort, there are still rare cases in which the diagnosis cannot be achieved with certainty.

## Data Availability

The datasets generated during the current study are available from the corresponding author on reasonable request.

## Abbreviations

GIT: Gastrointestinal tract
IHC: Immunohistochemical
LAMN: Low grade appendiceal mucinous neoplasm
MA: Mucinous cystadenoma
MBT: Mucinous borderline tumor
MC: Mucinous ovarian carcinoma
MeSH: Medical Subject Headings
NPV: Negative predictive value
PMP: Pseudomyxoma peritone
PPV: Positive predictive value
SEER: Surveillance Epidemiologic and End Results
